# Red and processed meat consumption and risk of pancreatic cancer: meta-analysis of prospective studies

**DOI:** 10.1038/bjc.2011.585

**Published:** 2012-01-12

**Authors:** S C Larsson, A Wolk

**Affiliations:** 1 Division of Nutritional Epidemiology, National Institute of Environmental Medicine, Karolinska Institutet, Box 210, SE-171 77 Stockholm, Sweden

**Keywords:** diet, meat, meta-analysis, pancreatic cancer, prospective studies, review

## Abstract

**Background:**

Whether red and processed meat consumption is a risk factor for pancreatic cancer remains unclear. We conducted a meta-analysis to summarise the evidence from prospective studies of red and processed meat consumption and pancreatic cancer risk.

**Methods:**

Relevant studies were identified by searching PubMed and EMBASE databases through November 2011. Study-specific results were pooled using a random-effects model.

**Results:**

Eleven prospective studies, with 6643 pancreatic cancer cases, were included in the meta-analysis. An increase in red meat consumption of 120 g per day was associated with an overall relative risk (RR) of 1.13 (95% confidence interval (CI)=0.93–1.39; *P*_heterogeneity_<0.001). Red meat consumption was positively associated with pancreatic cancer risk in men (RR=1.29; 95% CI=1.08–1.53; *P*_heterogeneity_=0.28; five studies), but not in women (RR=0.93; 95% CI=0.74–1.16; *P*_heterogeneity_=0.21; six studies). The RR of pancreatic cancer for a 50 g per day increase in processed meat consumption was 1.19 (95% CI=1.04–1.36; *P*_heterogeneity_=0.46).

**Conclusion:**

Findings from this meta-analysis indicate that processed meat consumption is positively associated with pancreatic cancer risk. Red meat consumption was associated with an increased risk of pancreatic cancer in men. Further prospective studies are needed to confirm these findings.

Pancreatic cancer is one of the most fatal types of cancer, with a 5-year relative survival of about 5.5% (Howlader *et al*, 2010). Thus, identification of risk factors for this cancer is of great public health importance. Dietary factors could conceivably influence the risk of developing pancreatic cancer, although no dietary factor has been convincingly associated with pancreatic cancer risk (2007). High consumption of red meat and/or processed meat has been associated with increased risk of some gastrointestinal cancers, such as colorectal (Larsson and Wolk, 2006; Chan *et al*, 2011) and stomach cancer (Larsson *et al*, 2006b). Whether red and processed meat consumption is a risk factor also for pancreatic cancer remains unclear. We therefore conducted a dose–response meta-analysis of prospective studies to examine the associations of red and processed meat consumption with pancreatic cancer risk.

## Materials and methods

### Search strategy and study selection

To identify prospective studies of red and processed meat consumption and pancreatic cancer risk, we conducted a literature search in PubMed and EMBASE databases for articles published in any language from January 1966 through November 2011. The following search terms were used: ‘meat’ or ‘foods’ and ‘pancreatic cancer’ or ‘pancreatic neoplasm’, and ‘cohort’ or ‘prospective’, or ‘nested case–control’. In addition, we searched the reference lists of retrieved articles to identify further studies.

To be included in our meta-analysis, studies had to (1) have a prospective design and with pancreatic cancer incidence or mortality as the outcome; and (2) provide relative risks (RRs) with 95% confidence intervals (CI) of pancreatic cancer for at least three categories (or as a continuous variable) of red meat and/or processed meat consumption.

### Data extraction

The following data were extracted from each publication: the first author's last name, year of publication, country in which the study was performed, sex, age, sample size, duration of follow-up, variables adjusted for in the multivariable model, and the RRs with CIs for each category of meat consumption. From each study, we extracted the RRs that reflected the greatest degree of control for potential confounders.

### Statistical analysis

Relative risks from individual studies and corresponding s.e. (derived from the CIs) were transformed to their natural logarithms to stabilise the variance and normalise the distributions. We used the method proposed by Greenland and Longnecker (1992) and Orsini *et al* (2006) to compute the trend from the correlated log RRs across categories of meat consumption. This method requires that the distribution of cases and person-time (or number of participants), and the RR with its variance estimate for at least three quantitative exposure categories be known. When meat consumption was expressed in ‘servings’ or ‘times’, we rescaled the consumption to grams per day using 120 g per day as the standard portion size for total and fresh red meat and 50 g as the standard portion size for processed meat (Norat *et al*, 2002). For each study, the median or mean level of consumption for each consumption category was assigned to each corresponding RR. When the median or mean consumption per category was not reported in the article, we assigned the midpoint of the upper and lower boundaries in each category as the average consumption. If the upper or lower boundary of the highest or lowest category was not provided, we assumed that it had the same amplitude as the closest category. If the amount of red meat per category was not specified in the article (Zheng *et al*, 1993; Coughlin *et al*, 2000; Isaksson *et al*, 2002), we estimated the amount using information from another article on meat consumption and disease in the same study population (Hsing *et al*, 1998; Chao *et al*, 2005; Rodriguez *et al*, 2006) or in a similar population with the same exposure (Stolzenberg-Solomon *et al*, 2002). We used an increase in red and processed meat consumption of 120 and 50 g per day, respectively, which corresponds to about a standard serving. We combined the RRs from each study by the method of DerSimonian and Laird (1986), using the assumptions of a random effects model, which takes into account both within- and between-study variability. We checked for nonlinearity of the dose–response relationship between meat consumption and pancreatic cancer by estimating polynomial models.

Statistical heterogeneity among study results was investigated using the *I*^2^-statistics (Higgins and Thompson, 2002). We conducted analyses stratified by geographical area (United States and Europe) and sex. Publication bias was examined with Egger's regression test (Egger *et al*, 1997). All statistical analyses were conducted with Stata (StataCorp, College Station, TX, USA). *P*-values were two-sided and *P*<0.05 was considered statistically significant.

## Results

### Study characteristics

We identified 13 prospective studies (Mills *et al*, 1988; Hirayama, 1989; Zheng *et al*, 1993; Coughlin *et al*, 2000; Isaksson *et al*, 2002; Stolzenberg-Solomon *et al*, 2002, 2007; Michaud *et al*, 2003; Nöthlings *et al*, 2005; Lin *et al*, 2006; Larsson *et al*, 2006a; Heinen *et al*, 2009; Inoue-Choi *et al*, 2011) that were potentially eligible for inclusion in the meta-analysis. Two studies were excluded, because the exposure was total meat, including white meat (poultry and fish; Mills *et al*, 1988), or the article was a review about the epidemiology of pancreatic cancer in Japan (Hirayama, 1989). The remaining 11 studies (Zheng *et al*, 1993; Coughlin *et al*, 2000; Isaksson *et al*, 2002; Stolzenberg-Solomon *et al*, 2002, 2007; Michaud *et al*, 2003; Nöthlings *et al*, 2005; Lin *et al*, 2006; Larsson *et al*, 2006a; Heinen *et al*, 2009; Inoue-Choi *et al*, 2011) were eligible for inclusion in the meta-analysis. Among these studies, six were carried out in the United States, four in Europe, and one in Japan (Table 1). The study population consisted of men and women in six studies: of only women in three studies, and of only men in two studies. Sample sizes ranged from 17 633–1 102 308, and the number of pancreatic cancer cases varied from 57 to 3751. Combined, these studies involved 6643 pancreatic cancer cases and a total of 2 307 787 participants. All studies adjusted for age and smoking, and most studies also adjusted for energy intake (*n*=7). Fewer studies controlled for body mass index (*n*=2) and/or history of diabetes (*n*=5).

### Red meat

**Figure 1:**
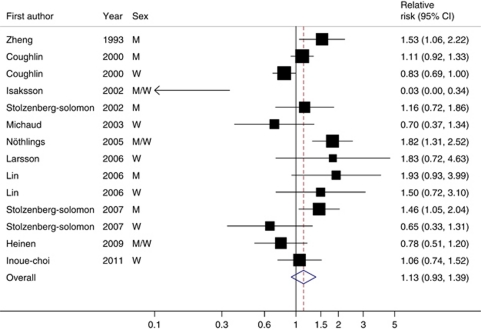
Relative risks of pancreatic cancer for a 120 g per day increase of red meat consumption. Squares indicate study-specific relative risks (size of the square reflects the study-specific statistical weight, i.e., the inverse of the variance); horizontal lines indicate 95% CIs; diamond indicates the summary relative risk estimate with its 95% CI. Test for heterogeneity: *Q*=43.05, *P*<0.001, *I*^2^=69.8%. All statistical tests were two-sided.

Eleven studies examined the association between consumption of fresh red meat (Michaud *et al*, 2003; Nöthlings *et al*, 2005; Lin *et al*, 2006; Larsson *et al*, 2006a; Heinen *et al*, 2009), pork (Isaksson *et al*, 2002), or total red meat (including processed meat; Zheng *et al*, 1993; Coughlin *et al*, 2000; Stolzenberg-Solomon *et al*, 2002, 2007; Inoue-Choi *et al*, 2011) and risk of pancreatic cancer. The RRs of pancreatic cancer associated with an increase of 120 g per day of red meat consumption are shown in Figure 1. We found no evidence of a non-linear association (*P* for nonlinearity=0.13). The overall RR indicated no statistically significant association between red meat consumption and pancreatic cancer (RR=1.13; 95% CI=0.93–1.39). There was statistically significant heterogeneity among studies (*P*<0.001; *I*^2^=69.8%). In a sensitivity analysis in which we removed one study at a time and analysed the rest, the RRs ranged from 1.08 (95% CI=0.89–1.31) after excluding the study by Nöthlings *et al* (2005) to 1.17 (95% CI=0.95–1.45) after excluding the study by Heinen *et al* (2009).

In stratified analysis, a statistically significant positive association between red meat consumption and risk of pancreatic cancer was observed in men (RR=1.29; 95% CI=1.08–1.53; *P*_heterogeneity_=0.28; five studies), but no association in women (RR=0.93; 95% CI=0.74–1.16; *P*_heterogeneity_=0.21; six studies). No association was observed in studies conducted in the United States (RR=1.13; 95% CI=0.90–1.42; *P*_heterogeneity_<0.001) or in Europe (RR=0.87; 95% CI=0.43–1.76; *P*_heterogeneity_=0.01). We found no evidence of publication bias (*P*=0.98).

### Processed meat

**Figure 2:**
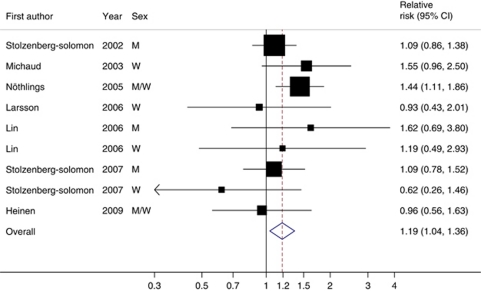
Relative risks of pancreatic cancer for a 50 g per day increase of processed meat consumption. Squares indicate study-specific relative risks (size of the square reflects the study-specific statistical weight, i.e., the inverse of the variance); horizontal lines indicate 95% CIs; diamond indicates the summary relative risk estimate with its 95% CI. Test for heterogeneity: *Q*=7.77, *P*=0.46, *I*^2^=0%. All statistical tests were two-sided.

Seven studies provided results for processed meat consumption (Stolzenberg-Solomon *et al*, 2002, 2007; Michaud *et al*, 2003; Nöthlings *et al*, 2005; Lin *et al*, 2006; Larsson *et al*, 2006a; Heinen *et al*, 2009). There was no evidence of a non-linear association between processed meat consumption and pancreatic cancer (*P* for nonlinearity=0.75). When results from all studies were combined, an increase of 50 g per day of processed meat consumption was associated with a statistically significant 19% increased risk of pancreatic cancer (RR=1.19; 95% CI=1.04–1.36), without heterogeneity among studies (*P*=0.46; *I*^2^=0%) (Figure 2). In a sensitivity analysis excluding one study at a time and analysing the rest, the RRs ranged from 1.11 (95% CI=0.95–1.30) to 1.24 (95% CI=1.05–1.46) after excluding the study by Nöthlings *et al* (2005) and Stolzenberg-Solomon *et al* (2002), respectively.

In analysis stratified by sex, the overall RRs were 1.11 (95% CI=0.92–1.34; *P*_heterogeneity_=0.68; three studies) in men and 1.12 (95% CI=0.75–1.67; *P*_heterogeneity_=0.29; four studies) in women. There was no statistically significant association between processed meat consumption and pancreatic cancer in studies conducted in the United States (RR=1.25; 95% CI=0.96–1.62; *P*_heterogeneity_=0.17; three studies) or Europe (RR=1.06; 95% CI=0.86–1.30; *P*_heterogeneity_=0.85; three studies), possibly because of limited statistical power. No publication bias was detected (*P*=0.53).

## Discussion

This meta-analysis showed a statistically significant positive association between processed meat consumption and risk of pancreatic cancer. An increase in processed meat consumption of 50 g per day, about one serving, was associated with a 19% increased risk of pancreatic cancer. The positive association between processed meat consumption and pancreatic cancer risk was attenuated and not statistically significant in a sensitivity analysis excluding one of the studies (Nöthlings *et al*, 2005). There was no overall association between red meat consumption and risk of pancreatic cancer. However, red meat consumption was statistically significantly positively associated with pancreatic cancer risk in men. Red meat consumption was on average higher in men than in women. If there is a threshold effect with an increased risk of pancreatic cancer only at very high levels of red meat consumption, a positive association may be more likely to be detected in men. The observed positive association in men may also be a chance finding.

Our study has some limitations. First, as a meta-analysis of observational studies, we cannot rule out that individual studies may have failed to control for potential confounders, which may introduce bias in an unpredictable direction. All studies controlled for age and smoking, but only a few studies adjusted for other potential confounders such as body mass index and history of diabetes. Another limitation is that our findings were likely to be affected by imprecise measurement of red and processed meat consumption and potential confounders. Categorisation of main exposures and confounders that are measured with error may induce misclassification and may bias the expected RR toward or away from the null value (Flegal *et al*, 1991; Wacholder *et al*, 1991; Wacholder, 1995). Thus, misclassification of red and processed meat consumption and of potential confounders might have resulted in an over- or underestimation of the association between red and processed meat consumption and risk of pancreatic cancer. Finally, publication bias could be of concern in meta-analysis. Nevertheless, we found no evidence of publication bias.

We excluded one study from this meta-analysis because the exposure was total meat including poultry and fish, and only one RR (for high *vs* low intake) was reported (Mills *et al*, 1988). That study included only 40 pancreatic cancer deaths and therefore would not have influenced the overall RRs if the study had been included. In that study, high consumption of total meat was associated with a RR of pancreatic cancer of 2.26 (95% CI=0.72–7.12; Mills *et al*, 1988).

A positive association between processed meat consumption and risk of pancreatic cancer is biologically plausible. Processed meats are usually preserved with nitrite and may also contain *N*-nitroso compounds. *N*-nitroso compounds can further be formed endogenously in the stomach from nitrite and ingested amides in foods of animal origin (Sen *et al*, 2000). *N*-nitroso compounds reach the pancreas via the bloodstream and are potent carcinogens that have been shown to induce pancreatic cancer in animal models (Risch, 2003). A population-based case–control study observed that intake of dietary nitrite from animal sources was statistically significantly positively associated with risk of pancreatic cancer in both men and women (highest *vs* lowest quartile odds ratio=2.3; 95% CI=1.1–5.1, for men and odds ratio=3.2; 95% CI=1.6–6.4, for women; Coss *et al*, 2004). A prospective study found that men in the highest quintile of summed nitrate/nitrite intake from processed meat had a nonsignificantly elevated risk of pancreatic cancer (hazard ratio=1.18, 95% CI=0.95–1.47; Aschebrook-Kilfoy *et al*, 2011).

Besides processed meat consumption, humans are exposed to *N*-nitroso compounds via cigarette smoking, which is an established risk factor for pancreatic cancer (Risch, 2003). Given that the main route of human exposure to *N*-nitroso compounds is cigarette smoke, the relation between processed meat consumption and pancreatic cancer risk may be modified by smoking status. Lin *et al* (2006) examined the association between ham and sausage consumption and risk of pancreatic cancer by smoking status, but observed no statistically significant association in neither smokers (highest *vs* lowest category RR=1.44; 95% CI=0.45–4.63) nor in nonsmokers (corresponding RR=1.16; 95% CI=0.43–3.19). However, the number of cases in the highest categories was very limited (⩽4 cases).

In conclusion, results from this meta-analysis indicated a statistically significant positive association between processed meat consumption and risk of pancreatic cancer. Red meat consumption was not associated with risk of pancreatic cancer overall, but was positively associated with risk in men. Large prospective studies with better adjustment for potential confounders are warranted to establish potential associations of red and processed meat consumption with pancreatic cancer risk. Whether the association between processed meat consumption and pancreatic cancer is modified by smoking needs further study.

## Figures and Tables

**Table 1:** Characteristics of prospective studies of red and processed meat consumption and pancreatic cancer risk^a^

**Study, country**	**Sample size, sex and age**	**No. of cases**	**Years of follow-up**	**Type of meat**	**RR (95% CI), highest *vs* lowest category**	**Adjustments**
Zheng *et al* (1993), United States	17 633 men, ⩾35 years	57	20	Red meat^a^	2.4 (1.0–6.1)	Age, smoking index, intakes of energy and alcohol
Coughlin *et al* (2000), United States	1 102 308 men and women, ⩾30 years	3751 (1967 men, 1784 women)	14	Red meat^a^ Red meat^a^	1.1 (0.9–1.2) men 0.9 (0.8–1.0) women	Age, race, smoking history, education, family history of pancreatic cancer, history of gallstones, history of diabetes, BMI, intakes of alcohol, citrus fruits and vegetables
Isaksson *et al* (2002), Sweden	21 884 men and women, NA	176	16	Pork	0.25 (0.08–0.81)	Age, sex, smoking, BMI
Stolzenberg-Solomon *et al* (2002), Finland	26 948 men, 50–69 years	163	13	Red meat^a^ Processed meat	0.95 (0.58–1.56) 1.04 (0.66–1.65)	Age, years of smoking and energy intake
Michaud *et al* (2003), United States	88 802 women, 30–55 years	178	18	Beef, pork or lamb Processed meat	0.75 (0.41–1.40) 1.28 (0.86–1.92)	Age, pack years of smoking, BMI, height, history of diabetes, energy intake
Nöthlings *et al* (2005), United States	190 545 men and women, 45–75 years	482	7	Beef, pork, or lamb Processed meat	1.45 (1.19–1.76) 1.68 (1.35–2.07)	Age, sex, ethnicity, smoking status, history of diabetes, family history of pancreatic cancer and energy intake
Larsson *et al* (2006a), Sweden	61 433 women, 40–76 years	172	15.3	Beef, pork, or veal Processed meat	1.73 (0.99–2.98) 0.94 (0.61–1.44)	Age, education, smoking status and pack years of smoking, BMI, and intakes of total energy, alcohol and folate
Lin *et al* (2006), Japan	105 438 men and women, 40–79 years	222 (106 men, 116 women)	9.9	Beef and pork^b^ Beef and pork^b^ Ham and sausage Ham and sausage	1.92 (0.95–3.86)^b^ men 1.56 (0.70–3.47)^b^ women 1.82 (0.62–4.26) men 0.93 (0.29–2.99) women	Age, area and pack years of smoking
Stolzenberg-Solomon *et al* (2007), United States	537 302 men and women, 50–71 years	836 (555 men, 281 women)	5	Red meat^a^ Red meat^a^ Processed meat Processed meat	1.42 (1.05–1.91) men 0.69 (0.45–1.05) women 1.07 (0.80–1.43) men 0.78 (0.48–1.12) women	Age, education, race, smoking, BMI, history of diabetes and intakes of energy and saturated fat
Heinen *et al* (2009), The Netherlands	120 852 men and women, 55–69 years	350	13.3	Fresh red meat Processed meat	0.75 (0.52–1.09) 0.93 (0.65–1.35)	Age, sex, smoking status and number of cigarettes smoked per day and number of years, BMI, history of diabetes, history of hypertension, intakes of energy, alcohol, vegetables and fruits
Inoue-Choi *et al* (2011), United States	34 642 women, 55–69 years	256	16.3	Red meat^b^	0.97 (0.65–1.44)	Age, race, education, smoking, physical activity and alcohol intake

Abbreviations: BMI=body mass index; CI=confidence interval; NA=not available; RR=relative risk (rate ratio or hazard ratio).

a Including processed meat.

b Results for beef and pork were combined using a random effects model.
